# Adjacency Matrix-Based Transmit Power Allocation Strategies in Wireless Sensor Networks

**DOI:** 10.3390/s90705390

**Published:** 2009-07-09

**Authors:** Luca Consolini, Paolo Medagliani, Gianluigi Ferrari

**Affiliations:** 1 Department of Information Engineering, University of Parma, viale G.P. Usberti 181/A, Parma, Italy; E-Mail: luca.consolini@unipr.it; 2 Wireless Ad-hoc and Sensor Networks (WASN) Laboratory, Department of Information Engineering, University of Parma, viale G.P. Usberti 181/A, Parma, Italy; E-Mail: gianluigi.ferrari@unipr.it

**Keywords:** adjacency matrix, power allocation, Zigbee wireless sensor network, performance, delay, network transmission rate, packet error rate, network connectivity, network lifetime

## Abstract

In this paper, we present an innovative transmit power control scheme, based on optimization theory, for wireless sensor networks (WSNs) which use carrier sense multiple access (CSMA) with collision avoidance (CA) as medium access control (MAC) protocol. In particular, we focus on schemes where several remote nodes send data directly to a common access point (AP). Under the assumption of finite overall network transmit power and low traffic load, we derive the optimal transmit power allocation strategy that minimizes the packet error rate (PER) at the AP. This approach is based on modeling the CSMA/CA MAC protocol through a finite state machine and takes into account the network *adjacency matrix*, depending on the transmit power distribution and determining the network connectivity. It will be then shown that the transmit power allocation problem reduces to a convex constrained minimization problem. Our results show that, under the assumption of low traffic load, the power allocation strategy, which guarantees minimal delay, requires the maximization of network connectivity, which can be equivalently interpreted as the maximization of the number of non-zero entries of the adjacency matrix. The obtained theoretical results are confirmed by simulations for unslotted Zigbee WSNs.

## 1. Introduction

Wireless sensor networks (WSNs) are an interesting research topic, both in military [1–3] and civilian scenarios [4]. In particular, remote/environmental monitoring, surveillance of reserved areas, etc., are important fields of application of WSNs. These applications often require very low power consumption and low-cost hardware [5]. One of the most common standards for wireless networking with low transmission rate and high energy efficiency has been proposed by the Zigbee Alliance [6]. In this context, an interesting research direction for WSNs is the design of network architectures that can guarantee high energy efficiency. In particular, since the overall energy available in a WSN is typically limited (all nodes are battery-equipped), the research community has focused on the derivation of transmit power allocation strategies that maximize a specific performance indicator yet still guarantee high energy savings.

In [7], the authors compare three power control schemes by analyzing the received signal-to-noise ratio in dense relay networks. In particular, one of these opportunistic schemes aims at extending the lifetime of the relays, in order to maximize the lifetime of the entire network. In [8], the authors introduce a power allocation scheme that minimizes the estimation mean-square error at the fusion center of a network where sensors transmit to the fusion center over noisy wireless links. In [9], the authors jointly optimize the data source quantization at each sensor, the routing scheme and the power control strategy in a WSN in order to derive an efficient solution for the problem of overall network optimization. Finally, in [10] the authors present an opportunistic power allocation strategy based on local and decentralized estimation of the links’ quality. In this scenario, only the nodes that experience channel conditions above a specific quality threshold are allowed to transmit in order to avoid waste of energy. In [11], the authors introduce a dynamic power allocation scheme for WSNs which relates the received signal strength indicator (RSSI) to the received signal-to-interference plus noise ratio (SINR). In particular, they propose two possible approaches: (i) a first approach based on a Markov chain system characterization and (ii) a second approach based on the minimization of the average packet error rate (PER).

In this paper, we propose an innovative transmit power control scheme for Zigbee WSNs based on optimization theory. This approach relies on the assumptions of (i) low traffic load and (ii) finite overall network transmit power, and it aims at the minimization of the PER at the access point (AP). Modeling the carrier sense multiple access with collision avoidance (CSMA/CA) medium access control (MAC) protocol through a finite state machine, it is possible to allocate the transmit powers at the sensors in order to maximize the number of 1’s of the adjacency matrix, i.e., the number of active pairwise connections between the nodes in the network — a 0 in an entry of the adjacency matrix indicates that the nodes corresponding to the row and the column are not connected. In all cases, we will assume that the sensors transmit directly to the AP. The proposed optimization approach will guarantee a lower PER than that in a scenario where all nodes transmit at the same power, yet still guarantee relevant energy savings.

The structure of this paper is the following. In Section 2., the analytical model, upon which this work is based, is presented. A simplified model is then derived, together with the network lifetime characterization and the optimized transmit power allocation strategy. In Section 3., the Zigbee standard and its implementation in the Opnet simulator are described. In Section 4., the performance, in terms of PER, delay and network lifetime, is presented, focusing on the impact of the adjacency matrix structure, the traffic load and the used power allocation strategy. Finally, Section 5. concludes this paper.

## 2. Analytical Model

### 2.1. Definition of a Simplified Model for Zigbee WSNs

In the following, we first introduce some key parameters of a Zigbee WSN. Then, we present a simplified version of its MAC protocol and under the assumption of low traffic load, we propose a simplified analytical model for the estimation of the following main network performance indicators: PER at the AP and average delay.

First of all, each sensor node is characterized by two main parameters: (i) its position on a two-dimensional plane and (ii) its transmit power, as stated in the following definition (for the sake of simplicity, we will simply use the term “sensor” to refer to a wireless node with sensing capabilities).

**Definition 1** *A* sensor *is represented by a couple s = (x, P), where x ∈* 𝕉^2^ *is the sensor position and P ∈ 𝕉 is its transmit power.*

We remark that the previous definition is based on the assumption that the positions of the nodes are known. This is realistic in several practical applications, such as industrial or home monitoring, where the spatial distribution of the nodes is *a priori* determined. In more general scenarios, the positions of the nodes could be unknown. In such case, one should also consider proper localization algorithms. However, once the positions of the nodes are estimated, our framework for optimized transmit power control can be directly applied.

We assume that the detection operation is described by an ideal threshold model, as stated in the following assumption.

**Assumption 1 (Threshold reception)** *Given two sensors s*_1_ = (*x*_1_, *P*_1_) *and s*_2_ = (*x*_2_, *P*_2_), *there exists a* minimum power function Π(*x*_1_, *x*_2_) *such that sensor s*_2_ *receives the transmission of sensor s*_1_ *if and only if*

$$P_{1}\geq \Pi \left(x_{1},x_{2}\right)$$

This assumption holds because of propagation loss (according to the Friis formula) and assumes that a threshold detector is used at the receiver [12]. In fact, in this case the power *P*_r_ received by sensor *s*_2_ can be expressed as:

(1) $$P_{r}=P_{1}G_{t}G_{r}\;{\left(\frac{\lambda }{4\pi r}\right)}^{\alpha }$$

where *G*_t_ and *G*_r_ are the gains of the transmit and receive antennas, *r* is the distance, λ is the wavelength, and *α* is the path loss exponent. According to the ideal threshold detector model, sensor *s*_2_ receives a transmission from sensor *s*_1_ if and only if *P_r_ > P*_min_, where *P*_min_ is the (pre-defined) receiver reception threshold. In this case,

$$\Pi \left(x_{1},x_{2}\right)=\frac{P_{\text{min}}}{G_{t}G_{r}}{\left(\frac{4\pi r}{\lambda }\right)}^{\alpha }$$

A sensor network can be introduced as a set of sensors, characterized by their positions and their transmit powers, together with an associated minimum power function.

**Definition 2** *A* sensor network *of N elements is an ordered set 𝒮* = (*c,* Π, *s*_1_, *s*_2_, . . ., *s_N_*), *where s*_1_, *s*_2_, . . ., *s_N_ are sensors, c ∈* 𝕉^2^ *is the position of the AP, and* Π : 𝕉^2^ ×𝕉^2^ → 𝕉 *is the associated minimum power function.*

**Definition 3 (Adjacency matrix)** *Given a sensor network 𝒮* = (*c*, Π, *s*_1_, *s*_2_, . . ., *s_N_*), *with s_i_* = (*x_i_*, *P_i_*), *i* = 1, . . ., *N, its associated* adjacency matrix *is given by*

$$A(𝒮)\in {\mathbb{R}}^{N\times N}$$

*where*

$$A_{\mathrm{ij}}=A{\left(𝒮\right)}_{\mathrm{ij}}=\{\begin{array}{ll} 1\; & \;\mathrm{if}\;P_{i}\geq \Pi \left(x_{i},x_{j}\right)\\ 0 & \mathrm{otherwise}. \end{array}$$

*The complement of A(𝒮) corresponds to*

$$\bar{A}\left(𝒮\right)=\{\begin{array}{ll} 1\; & \;\mathrm{if}\;A_{\mathrm{ij}}=0\\ 0 & \mathrm{otherwise}. \end{array}$$

*The number of ones in the adjacency matrix is given by the* adjacency *of sensor network 𝒮 and denoted by* |*A*(*𝒮*)|. *The complementary adjacency is given by the number of zeros in the adjacency matrix and denoted by* |*Ā*(*𝒮*)|.

*For each i* = 1, . . ., *N, we define the following two sets:*

$$\begin{array}{c} {𝒭}_{i}\;\;≜\;\;\;\left\{j=1,\ldots ,N|A_{\mathrm{ji}}=1\right\}\\ {𝒯}_{i}\;\;≜\;\;\;\left\{j=1,\ldots ,N|A_{\mathrm{ij}}=1\right\} \end{array}$$

*which represent the sets of indices of the sensors that s_i_ can receive from and transmit to, respectively. We denote by 𝒭̄_i_ and 𝒯̄_i_ the complements of these two sets.*

In order to make the theoretical analysis feasible, a Zigbee WSN is described by the following simplified model.

## Assumption 2 (Simplified model)

1. Poisson generation: the traffic generated by each sensor in the network is modeled as a homogeneous Poisson process [13]. The processes associated with different sensors are independent of each other and have intensity g (dimension: [pck/s]) [14].
2. *Limited CCA: before transmission, the i-th sensor waits for a random backoff time, with average T*_B_1__ *(dimension: [s]), and then checks if the channel is clear. This clear channel assessment (CCA) is limited only to those sensors whose indices lie in the set 𝒭_i_. In other words, the sensing is limited only to those sensors that can effectively (i.e., with sufficiently high received power) transmit to the i-th sensor. The CCA has a duration equal to T*_CCA_.
3. *Infinite number of backoffs: if the channel is found busy, the current sensor transmission is delayed by a random backoff time with average T*_B_2__ *(dimension: [s]). During the backoff period the traffic generation at the transmitting sensor does not stop. There is no limit on the total number of subsequent backoffs that a single packet transmission can incur.*
4. *Constant transmission length: each transmission has the same length T*_trans_ *= L/R, where L is the packet length (dimension: [b/pck]) and R is the transmission data rate (dimension: [b/s]).*
5. *Transmission turnaround time: after sensing, if the channel is found idle, each sensor waits a turnaround time, denoted as T*_TAT_ *(dimension: [s]), before starting its transmission.*

For each sensor *s_i_* (*i* = 1, . . ., *N*) the following counting processes can be defined.

- *G_i_*(*t*): the number of times that sensor *s_i_* has checked if the channel is clear in the time interval [0, *t*].
- *B_i_*(*t*): the number of times that a packet transmission of sensor *s_i_* has been delayed, through the backoff mechanism, in [0, *t*].
- *T_i_*(*t*): the number of times that a sensor *s_i_* has transmitted in [0, *t*] (counting both successful and unsuccessful transmissions).
- *E_i_*(*t*): the number of transmission errors incurred by sensor *S_i_* in [0, *t*].

For a counting process *P*(*t*), define the steady state intensity as follows:

(2) $$F[P]≜\underset{t\to \infty ,\tau \to 0}{\text{lim}}\frac{𝔼[P(t+\tau )-P(t)]}{\tau }$$

where 𝔼[·] denotes expectation. We recall that for the stationary Poisson traffic generation processes, the steady state intensity is constant and denoted by *g*. In the following, we assume that the limit at the righthand side of Equation 2 exists for all previously defined counting processes: this is equivalent to assuming that the network reaches a “steady state.” Under this hypothesis, the following equilibrium conditions must be satisfied:

(3) $$F\left[T_{i}\right]=g$$

(4) $$F\left[G_{i}\right]=g+F\left[B_{i}\right]$$

Equation 3 states that, at steady state, the intensity of transmissions must be equal to the intensity of traffic generation. Equation 4 states that, at steady state, the intensity of channel sensing has to be equal to the sum of the intensities of packet generations and backoffs.

The backoff traffic intensity can be expressed as follows:

$$F\left[B_{i}\right]=F\left[G_{i}\right]\chi_{i}$$

where *χ_i_* represents the ratio between the numbers of backoffs and transmission attempts. In this way, the processes {*T_i_*(*t*)} and {*B_i_*(*t*)} satisfy the following relations:

(5) $$F\left[G_{i}\right]=F\left[B_{i}\right]+F\left[T_{i}\right]=\frac{g}{1-\chi_{i}}$$

(6) $$F\left[B_{i}\right]=\frac{\chi_{i}}{1-\chi_{i}}g.$$

The term *χ_i_* can be equivalently interpreted as the probability, for the *i*th sensor, to assess that the channel is busy during the CCA. In order to derive a simple expression for *χ_i_*, it is assumed that the processes {*T_i_*(*t*)} are uncorrelated and Poisson. This simplification is appropriate under low traffic conditions. In fact, in this case, *F*[*B_i_*] << *g* and the processes {*T_i_*(*t*)} are statistically very similar to Poisson traffic generation processes. However, as it will be shown in Section 4., the estimated PER obtained with these simplifications is close to that predicted by (realistic) simulations also under relatively high traffic conditions.

Under the above simplifications, *χ_i_* equals the probability of finding at least one packet transmission event, during a time interval equal to the transmission length *T*_trans_, in the set of independent Poisson processes {*G_j_*(*t*)}*_j∈𝒭_i__*. In other words, one can write:

$$\chi_{i}=\underset{t\to \infty }{\text{lim}}𝒫\left\{\underset{j\in {𝒭}_{i}}{\text{max}}\left\{T_{j}\left[t+T_{\text{trans}}\right]-T_{j}\left[T_{\text{trans}}\right]\right\}>0\right\}$$

In order to compute *χ_i_*, it is worth remarking that the probability of finding no packet transmissions from the *i*th sensor in a time interval of length *T*_trans_ is given by *e*^−*F*[*T*_*i*_]*T*_trans_^. Since the process *T_i_* is assumed to be Poisson and uncorrelated from the other {*T_j_*}*_j≠i_*, the probability of finding no transmission events from the sensors belonging to *𝒭_i_* (i.e., those sensors that can be received by the *i*th sensor) in *T*_trans_ is given by

$$\prod_{j\in {𝒭}_{i}}\left(e^{-F\left[T_{j}\right]T_{\text{trans}}}\right)$$

In conclusion, the probability of finding at least one packet transmission event in a time interval equal to *T*_trans_ from any of the sensors that can be received by the *i*th sensor is given by

$$1-\prod_{j\in {𝒭}_{i}}\left(e^{-F\left[T_{j}\right]T_{\text{trans}}}\right)$$

Therefore, *χ_i_* can finally be expressed as follows:

(7) $$\begin{array}{lll} \chi_{i} & = & 1-\prod_{j\in {𝒭}_{i}}\left(1-e^{-F\left[T_{j}\right]T_{\text{trans}}}\right)\\ & ≃ & \sum_{j\in {𝒭}_{i}}gT_{\text{trans}}\\ & = & g\left|{𝒭}_{i}\right|T_{\text{trans}} \end{array}$$

where we have used Equation 3 and approximated Π_*j*∈*𝒭_i_*_ (1 − *e*^−*gT*_trans_^) with ∑_*j*∈*𝒭_i_*_ *gT*_trans_. The latter simplification holds under low traffic conditions, where *gT*_trans_ << 1. The notation |*𝒭_i_*| stands for the number of elements of the set *𝒭_i_*. From Equation 7, using the approximations 1/(1 − *χ_i_*) ≃ 1 + *χ_i_* and *χ_i_*/(1 − *χ_i_*) ≃ *χ_i_*, that hold for small values of *χ_i_*, the following simplified expressions for network sensing and backoff intensities can then be obtained:

$$\begin{array}{l} F\left[G_{i}\right]≃\left(1+\sum_{j\in {𝒭}_{i}}gT_{\text{trans}}\right)g\\ F\left[B_{i}\right]≃\left(\sum_{j\in {𝒭}_{i}}gT_{\text{trans}}\right)g \end{array}$$

In general, the number of transmission errors accumulated by sensor *s_i_* can be written in the following form:

(8) $$F\left[E_{i}\right]=\gamma_{i}F\left[G_{i}\right]+\lambda_{i}F\left[T_{i}\right]+\eta_{i}F\left[T_{i}\right]+\kappa_{i}F\left[T_{i}\right]$$

where the four terms at the righthand side can be characterized as follows. The term *γ_i_F*[*G_i_*] represents the intensity of transmission errors occurred due to the occupation of channel by a packet transmission that could not be detected by the *i*th sensor during a CCA interval. The term λ*_i_F*[*T_i_*] represents the intensity of transmission errors due to interference from other sensors that cannot receive *s_i_*. The term *η_i_F*[*T_i_*] represents the intensity of transmission errors resulted from another sensor beginning to transmit when *s_i_* is waiting the turnaround time between the CCA and the transmission act. Finally, the term *κ_i_F*[*T_i_*] represents the intensity of transmission errors due to the fact that other sensors can begin transmission in the first subinterval, of length *T*_TAT_, of a transmission act from sensor *s_i_*. In fact, if some other sensor begins transmission during the turnaround time, it cannot detect the previous starting instant of a transmission by *s_i_*. The last two terms appearing in Equation 8 take into account the transmission errors independent of the network connectivity and are significant in the overall network error analysis.

Under the assumption of low traffic load and with the simplification that all relevant processes are Poisson and independent, the coefficient *γ_i_* in Equation 8 can be approximated as follows:

$$\begin{array}{lll} \gamma_{i} & = & \underset{t\to \infty }{\text{lim}}𝒫\left\{\underset{j\in {\bar{𝒭}}_{i}}{\text{max}}\left\{T_{j}\left[t+T_{\text{trans}}\right]-T_{j}\left[T_{\text{trans}}\right]\right\}>0\right\}\\ & = & 1-\prod_{j\in {\bar{R}}_{i}}\left(1-e^{-F\left[T_{j}\right]T_{\text{trans}}}\right)\\ & ≃ & \sum_{j\in {\bar{𝒭}}_{i}}F\left[T_{j}\right]T_{\text{trans}}≃\left|{\bar{𝒭}}_{i}\right|T_{\text{trans}}\;g \end{array}$$

Similarly, the coefficient λ*_i_* in Equation 8 can be approximated as

$$\begin{array}{lll} \lambda_{i} & = & \underset{t\to \infty }{\text{lim}}𝒫\left\{\underset{j\in {\bar{𝒯}}_{i}}{\text{max}}\left\{G_{j}\left[t+T_{\text{trans}}\right]-G_{j}\left[T_{\text{trans}}\right]\right\}>0\right\}\\ & = & 1-\prod_{j\in {\bar{𝒯}}_{j}}\left(1-e^{-F\left[G_{j}\right]T_{\text{trans}}}\right)\\ & ≃ & \sum_{j\in {\bar{𝒯}}_{j}}F\left[G_{j}\right]T_{\text{trans}}≃\left|{\bar{𝒯}}_{j}\right|T_{\text{trans}}\;g \end{array}$$

The coefficient *η_i_* in Equation 8 can be approximated as

$$\begin{array}{lll} \eta_{i} & = & \underset{t\to \infty }{\text{lim}}𝒫\left\{\underset{j=1,\ldots ,N}{\text{max}}\left\{T_{j}\left[t+T_{\text{TAT}}\right]-T_{j}\left[t\right]\right\}>0\right\}\\ & = & 1-\prod_{j=1,\ldots ,N}\left(1-e^{-F\left[G_{i}\right]T_{\text{TAT}}}\right)≃{\mathrm{NgT}}_{\text{TAT}} \end{array}$$

Finally, the coefficient *k_i_* in Equation 8 is given by

$$\begin{array}{lll} \kappa_{i} & = & \underset{t\to \infty }{\text{lim}}𝒫\left\{\underset{j=1,\ldots ,N}{\text{max}}\left\{T_{j}\left[t+T_{\text{TAT}}\right]-T_{j}\left[t\right]\right\}>0\right\}\\ & = & 1-\prod_{j=1,\ldots ,N}\left(1-e^{-F\left[T_{i}\right]T_{\text{TAT}}}\right)≃{\mathrm{NgT}}_{\text{TAT}} \end{array}$$

Using the expressions found above for the coefficients *γ_i_*, *λ_i_*, *η_i_* and *κ_i_* in Equation 8, the transmission error intensity can be approximated as

$$F\left[E_{i}\right]≃\left[\left(\left|{\bar{𝒯}}_{i}\right|+\left|{\bar{𝒭}}_{i}\right|\right)T_{\text{trans}}+{2\mathrm{NT}}_{\text{TAT}}\right]g^{2}$$

Therefore, the overall network error intensity can be estimated as follows:

$$\sum_{i=1}^{N}F\left[E_{i}\right]≃\left(2|\bar{A}(𝒮)|T_{\text{trans}}+2N^{2}T_{\text{TAT}}\right)g^{2}$$

and the error probability, i.e., the ratio between the overall network error intensity and the generation intensity (given by *Ng*), becomes

(9) $$\begin{array}{lll} P_{\text{er}} & = & \frac{\sum_{i=1}^{N}F\left[E_{i}\right]}{\mathrm{Ng}}\\ & ≃ & \left(2\frac{\left|\bar{A}(𝒮)\right|}{N^{2}}T_{\text{trans}}+{2T}_{\text{TAT}}\right)\mathrm{Ng} \end{array}$$

Equation 9 shows that, under the considered simplifying assumptions, the error probability grows linearly with the network complementary adjacency |*Ā*(*𝒮*)|.

In the following, we find an estimate of the average network delay. First of all, we remark that if, after the first backoff, the channel is found idle, the total delay is given by

$$D_{\text{min}}=T_{B_{1}}+T_{\text{CCA}}+T_{\text{TAT}}+T_{\text{trans}}$$

This is the minimum average delay that a packet incurs if the channel is found idle at the first transmission attempt. If the channel is found busy, the sensor waits for a backoff time with average T_B_2__, then senses the channel again. If, taking into account the second transmission attempt, the channel is found idle for the second time, the overall delay can be expressed as *D*_min_ + *D*_BO_, where

$$D_{\text{BO}}=T_{B_{2}}+T_{\text{CCA}}$$

Under the low traffic load assumption, the probability of having more than one backoff during a single transmission act is negligible. Therefore, the average transmission delay becomes

$$\begin{array}{lll} D_{i} & = & D_{\text{min}}+D_{\text{BO}}\gamma_{i}=D_{\text{min}}+D_{\text{BO}}\sum_{j\in {\bar{𝒭}}_{i}}F\left[T_{j}\right]T_{\text{trans}}\\ & ≃ & D_{\text{min}}+D_{\text{BO}}\left|{\bar{𝒭}}_{i}\right|{\mathrm{gT}}_{\text{trans}} \end{array}$$

where *D*_BO_ is the average backoff time. The average network delay can then be expressed as

(10) $$\begin{array}{lll} D & = & \frac{\sum_{i=1}^{N}D_{i}}{N}\\ & ≃ & D_{\text{min}}+D_{\text{BO}}\frac{\left|A(𝒮)\right|}{N}T_{\text{trans}}g. \end{array}$$

Equation 10 for the delay shows that the network delay depends linearly on the network adjacency. We remark that, since we considered star topologies, the PER and delay statistics collected at each node are less significant than those calculated at the AP, which instead provide a better description of the network behavior. Should more complicated topologies be considered, the proper metrics need to be taken into account.

In conclusion, under the low traffic load assumption, the PER and the delay at the AP of a WSN can be estimated as follows:

(11) $$\begin{array}{lll} P_{\text{er}} & ≃ & \left(2\frac{\left|\bar{A}(𝒮)\right|}{N^{2}}T_{\text{trans}}+{2T}_{\text{TAT}}\right)\mathrm{Ng}\\ D & = & T_{B_{1}}+T_{\text{CCA}}+T_{\text{TAT}}+\left(T_{B_{2}}+T_{\text{CCA}}\right)\left(T_{\text{trans}}\frac{\left|A(𝒮)\right|}{N}g\right) \end{array}$$

### 2.2. Network Lifetime

An important parameter for a WSN is the network lifetime. This performance indicator can be interpreted in several ways. For example, in [11] the network lifetime is defined as the time interval at the end of which the probability of outage falls below a maximum value than can be tolerated, on average, over the transmission links before the network is declared dead. In particular, the network degradation (i.e., the increase of the probability of outage) is assumed to be caused by fading and battery depletion. In [15], the network lifetime is related to the minimum number of sensors that need to be active before declaring the network dead. More precisely, when the number of active nodes drops to below this minimum number due to battery depletion, the network dies.

In this paper, the network lifetime is defined similarly to that proposed in [15]. More precisely, since this paper focuses on power control, i.e., minimization of the total transmit power for a given PER, we consider a definition of network lifetime based on the overall residual energy in the network. If the overall residual energy at time *t*, denoted as *E*_res−net_(*t*) is higher than a pre-defined threshold, which may depend on a required network operational quality of service (QoS), then the network is declared alive. On the other hand, if the residual energy becomes lower than this threshold, then the network is declared dead.

The network residual energy at time *t* can be expressed as:

$$E_{\text{res}-\text{net}}\;(t)=NE_{I-\text{node}}-E_{\text{cons}-\text{net}}\;(t)$$

where *E*_I−node_ is the initial per-node energy and *E*_cons−net_(*t*) is the average energy consumed, at network level, up to time *t*. In order to evaluate *E*_cons−net_(*t*), one can write:

(12) $$E_{\text{cons}-\text{net}}\;(t)=P_{\text{cons}-\text{net}}\;t=NP_{\text{cons}-\text{node}}\;t$$

where *P*_cons−net_ is the average network-level consumed power and *P*_cons−node_ is the average consumed power at each node. When the proposed power allocation strategy is used, the consumed power at each sensor is different. However, in order to simplify the analytical model, we consider the average network-wide power consumed, then we derive the average power consumed at each node. At this point, the evaluation of the average network residual energy at any instant reduces to the evaluation of *P*_cons−node_.

In order to evaluate *P*_cons−node_, one can observe that it depends on the average powers consumed by the nodes in each of the following possible states: (i) transmission (tx), (ii) reception (rx), (iii) CCA, (iv) BO, and (v) idle. We denote the average percentages of time, in 1 s, spent by the nodes in each of the previous states as (i) *τ*_tx_state_, (ii) *τ*_rx_state_, (iii) *τ*_CCA_state_, (iv) *τ*_Boff_state_, and (v) *τ*_idle_state_, respectively. The average power consumed at each node can then be evaluated as follows:

(13) $$\begin{array}{ccc} P_{\text{cons}-\text{node}} & = & \tau_{\text{tx}\_\text{state}}P_{\text{tx}\_\text{state}}+\tau_{\text{rx}\_\text{state}}P_{\text{rx}\_\text{state}}+\tau_{\text{CCA}\_\text{state}}P_{\text{CCA}\_\text{state}}\\ & & +\tau_{\text{Boff}\_\text{state}}P_{\text{Boff}\_\text{state}}+\tau_{\text{idle}\_\text{state}}P_{\text{idle}\_\text{state}} \end{array}$$

At this point, we simply need to evaluate (i) the percentages of time and (ii) the powers appearing at the right-hand side of Equation 13. We start with the percentages of time. As stated, we refer to the percentages of time spent in the various states within a 1 s interval. We remark that the assumption of a reference time equal to 1 s holds since *gT*_trans_ ≪ 1. In fact, if *gT*_trans_ ≥ 1, each node would always be in the transmission state, and the network would not function. Likewise, the other percentages of times are all lower than 1 under the assumed low traffic load conditions. The percentage of time spent by a node in the tx state can be computed as

$$\tau_{\text{tx}\_\text{state}}={\mathrm{gT}}_{\text{trans}}.$$

The percentage of time spent in the rx state for a generic node *i* can be computed as the sum of the transmission time percentages of the nodes which are within the transmission range of node *i*, i.e., from the nodes belonging to *𝒭_i_*. Owing to the previous derivations, this percentage of time does not depend on the particular node and, exploiting the results in Equations 5 and 6, can be expressed as follows:

$$\tau_{\text{rx}\_\text{state}\_i}=\sum_{j\in {𝒭}_{i}}{\mathrm{gT}}_{\text{trans}}={\mathrm{gT}}_{\text{trans}}\left|{𝒭}_{i}\right|={\mathrm{gT}}_{\text{trans}}\sum_{j=1}^{N}\left|A_{\mathrm{ji}}\right|$$

The percentage of time spent in the CCA phase can be evaluated as

$$\tau_{\text{CCA}\_\text{state}}=F\left[G_{i}\right]T_{\text{CCA}}≃\left(1+\sum_{j\in {𝒭}_{i}}{\mathrm{gT}}_{\text{trans}}\right)\;{\mathrm{gT}}_{\text{CCA}}=\left(1+{\mathrm{gT}}_{\text{trans}}\sum_{j=1}^{N}\left|A_{\mathrm{ji}}\right|\right)\;{\mathrm{gT}}_{\text{CCA}}$$

The fraction of time spent in the BO state by a generic node *i*, under the assumption that the node experiences only a single BO before transmitting a packet, can be written as

$$\tau_{\text{Boff}\_\text{state}}=F\left[B_{i}\right]T_{B_{1}}≃\left(\sum_{j\in {𝒭}_{i}}{\mathrm{gT}}_{\text{trans}}\right)\;{\mathrm{gT}}_{B_{1}}=\left({\mathrm{gT}}_{\text{trans}}\sum_{j=1}^{N}\left|A_{\mathrm{ji}}\right|\right)\;{\mathrm{gT}}_{B_{1}}$$

Finally, since the previous percentages of time have been evaluated with respect to a reference interval that equals to 1 s, the percentage of time spent by a node in the idle state can be expressed as

$$\tau_{\text{idle}\_\text{state}}=1-\left(\tau_{\text{tx}\_\text{state}}+\tau_{\text{rx}\_\text{state}}+\tau_{\text{CCA}\_\text{state}}+\tau_{\text{Boff}\_\text{state}}\right)$$

In order to evaluate the average power consumption in each state, we refer to the results presented in [16], where the authors evaluate the power consumption of a generic node equipped with a CC2420 radio. In particular, the current consumption in the tx state depends linearly on the transmit power. The power consumption in each state is shown in Table 1.

These terms have been obtained as a linear interpolation of the values presented in [16]. We point out that the dimension of the coefficient *P_i_* is 1/V. The voltage reference for the evaluation of the consumed power is *V*_DD_ = 3 V. Given the current consumption, it is possible to derive the associated power consumption by simply multiplying the current consumption by the reference voltage. In this way, the values of the powers in the various states (excluding the tx state) become:

(14) $$P_{\text{idle}\_\text{state}}=V_{\text{DD}}I_{\text{idle}\_\text{state}}=1.188\;\text{mW}$$

(15) $$P_{\text{rx}\_\text{state}}=V_{\text{DD}}I_{\text{rx}\_\text{state}}=58.8\;\text{mW}$$

(16) $$P_{\text{CCA}\_\text{state}}=V_{\text{DD}}I_{\text{CCA}\_\text{state}}=58.8\;\text{mW}$$

(17) $$P_{\text{Boff}\_\text{state}}=V_{\text{DD}}I_{\text{Boff}\_\text{state}}=1.188\;\text{mW}$$

The power consumed in the tx state can be expressed as the arithmetic average of the specific transmit powers used by all nodes in the network:

(18) $$P_{\text{tx}\_\text{state}}=\frac{\sum_{i=1}^{N}I_{\text{tx}\_\text{state}\_i}V_{\text{DD}}}{N}=\frac{\sum_{i=1}^{N}\left(23.598\;P_{i}+0.029133\right)}{N}$$

Note that the values of {*P_i_*} will be determined by the proposed power allocation strategy. Obviously, if a uniform power allocation strategy is used, i.e., {*P_i_*} are all equal, the proposed derivation still holds.

### 2.3. Optimal Transmit Power Allocation

In this subsection, we discuss the following problem:

**Problem 1 (Transmission error optimization)** *Upon the assignment of a total available transmit power P*_tot_ *for the sensor network 𝒮, distribute it among the sensors in the network in order to minimize the PER at the AP.*

This problem is equivalent to minimizing the overall transmit power to guarantee a desired PER at the AP.

Under low traffic load assumption, using Equation 11 on the PER, the solution of Problem 1 is equivalent to the maximization of the adjacency |*A*(*𝒮*)| of sensor network *𝒮*. This fact allows to recast Problem 1 in the following form.

**Problem 2 (Network adjacency maximization)** *Upon the assignment of a total available transmit power P*_tot_ *for the sensor network 𝒮, distribute it among the sensors in the network in order to maximize the network adjacency* |*A*(*𝒮*)|.

Assign to each sensor *s_i_* a transmission power *P_i_* > 0, *i* = 1, . . ., *N*. Then, the network adjacency is given by the following function:

(19) $$|A(𝒮)|=Q_{\text{tot}}\;\left(P_{1},P_{2},\ldots ,P_{N}\right)=\sum_{i=1,\ldots ,N,\;j=1,\ldots ,N}H\left(P_{i}-\Pi \left(x_{i},x_{j}\right)\right)$$

where

$$H(x)=\{\begin{array}{ll} 1 & \text{if}\;x\geq 0\\ 0 & \text{otherwise} \end{array}$$

is the Heaviside function. We remark that the Heaviside function *H*(*P_i_* − Π(*x_i_, x_j_*)) appearing in Equation 19 is 1 if the *i*th sensor transmits with a power sufficient to reach the *j*th sensor, and 0 if otherwise.

For each sensor *s_i_*, define *𝒫_i_* as the following set of transmit power values:

(20) $${𝒫}_{i}≜\left\{\Pi \left(x_{i},x_{j}\right),\;j=1,\ldots ,N,\;\;\text{with}\;j\neq i:\Pi \left(x_{i},x_{j}\right)\geq P_{\text{min},i}\right\}\cup \left\{P_{\text{min},i}\right\}$$

where Π(*x_i_, x_j_*) is the transmit power with which sensor *s_i_* can reach sensor *s_j_* and *P*_min*,i*_ is the minimum power that allows the *i*th sensor to reach the AP. According to this definition, the set *𝒫_i_* contains the value of the minimum transmit power required by the *i*th sensor to reach the AP, together with the values of the transmit powers that allow *s_i_* to reach the other sensors of the network and are higher than *P*_min*,i*_.

The following property leads to the possibility of limiting the search of possible transmit powers for a sensor *s_i_* to the set *𝒫_i_*.

**Proposition 1** *For any set of transmit powers P_i_ >* 0, *i* = 1, . . ., *N, there exists a set of values P̄_i_ ∈ 𝒫_i_, such that*

(21) $$Q_{i}\left({\bar{P}}_{1},{\bar{P}}_{2},\ldots ,{\bar{P}}_{N}\right)=Q_{i}\left(P_{1},P_{2},\ldots ,P_{N}\right),$$

(22) $${\bar{P}}_{i}\leq P_{i},\;\forall i=1,\ldots ,N$$

Proof.

Define

(23) $${\bar{P}}_{i}≜\text{max}\left\{P\in {𝒫}_{i}:P\leq P_{i}\right\}$$

Equation 22 follows immediately from Equation 23. Moreover, the function at the righthand side of Equation 19 is piecewise constant with respect to any argument *P_i_*, discontinuous on those values in which *P_i_* = Π(*x_i_, x_j_*) for any *j* = 1, . . ., *N*. From Equations 20 and 23 it follows that function *Q* is continuous in the set [*P̄*_1_, *P*_1_]×[*P̄*_2_, *P*_2_] … [*P̄_N_, P_N_*] and therefore constant in this set. Hence, Equation 21 holds.

Proposition 1 simply means that, in the ideal threshold detection hypothesis, it is not convenient to allocate to sensor *s_i_* a transmit power that does not belong to the set *𝒫_i_*, since it would employ extra power without gaining extra connectivity. For instance, in a network composed of 4 sensors, suppose that sensor 1 can reach the AP using a transmit power of 0.5 mW, whereas it needs 1 mW to reach sensor 2, 2 mW to reach sensor 3, and 0.2 mW to reach sensor 4, respectively. In this case, *𝒫_i_* = {0.5 mW, 1 mW, 2mW} contains the transmit powers that allow to reach the AP and sensors 2 and 3. The optimal transmit power for the first sensor should be chosen in this set. In fact, for example, it would be inconvenient to choose a transmit power of 1.5 mW instead of 1 mW, because the connectivity would be the same despite the increased transmit power (sensor 1 would still reach the AP and sensors 2 and 4).

The power allocation problem may be written in the following form.

**Problem 3 (Discrete optimization problem)** *For each sensor i* = 1, . . ., *N choose a transmit power P_i_ ∈ 𝒫_i_ such that the function T*(*P*_1_, *P*_2_, . . ., *P_N_*) *(defined by Equation 19) is maximized while satisfying the constraint*

$$\sum_{i=1}^{N}P_{i}\leq P_{\text{tot}}$$

This problem corresponds to a *multiple choice knapsack problem*, which has been extensively studied in the literature [17] and can be solved by standard computational libraries, such as MOSEK [18]. It is well known that this problem is NP-complete and the computation time increases very quickly as the number of sensors in the network grows. However, this is a standard optimization problem and some recent tools allow finding the exact solution in a reasonable time, in many cases of practical interest. Table 2 shows the computation time (namely the mean value and the standard deviation), in relation to the size of the sensor network, obtained with MOSEK 5 (64-bit version) running over a Core 2 Duo CPU with a clock frequency of 3.16 GHz and a 4 GB RAM. Furthermore, it is worth noting that accurate suboptimal solutions to problems of larger size (i.e., considering larger networks) could be obtained through heuristic methods.

**Figure 1.**
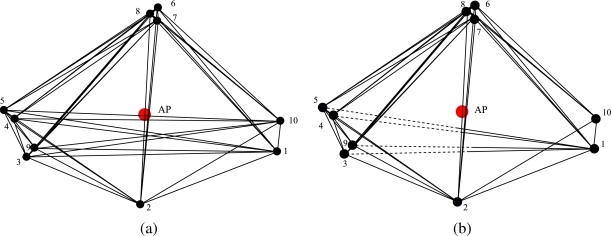
Pairwise connections in a scenario with *N* = 10 nodes (this scenario will correspond to that in Figure 4a). Two values for the total network transmit power are considered: (a) *P*_tot_ = 5 · 10*^−^*^5^ W and (b) *P*_tot_ = 2.5 · 10*^−^*^5^ W. In both cases, the proposed optimized power allocation strategy is used.

**Figure 2.**
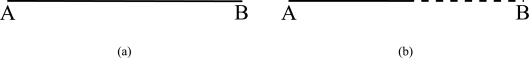
Graphical notation for communication links: (a) A and B communicate with each other (bidirectional communication); (b) only A can transmit to B (monodirectional communication).

An illustration of how the proposed approach works is depicted in Figure 1, whose legend is shown in Figure 2. When the transmit power budget is large enough to allow each node to communicate with any other node (Figure 1a), all bidirectional connections are active (solid lines, as shown in Figure 1a). When the power budget is not large enough (Figure 1b), the proposed optimized transmit power allocation strategy allocates the transmit power to the nodes in a way that the number of 1’s in the adjacency matrix is maximized. This means that some connections may be missing (absence of connecting lines between the nodes) or become monodirectional (half solid and half dashed lines, as shown in Figure 1b).

## 3. Simulation Model

### 3.1. Zigbee Standard

The increasing need for applications, where nodes can send data without the constraints imposed by the presence of power and transmission cables, have led to the creation of low-rate wireless personal area networks (LR-WPANs). This is the case, for example, of remote monitoring of natural events, such as landslides, earthquakes, etc. [19, 20]. One of the newest standards for WSNs, with significant power savings, is Zigbee [6]. More precisely, the Zigbee Alliance provides instructions only for the upper layers (i.e., from the third to the seventh layer) of the ISO/OSI stack [21]. At the first layers of the ISO/OSI stack (physical, PHY, and medium access control, MAC), the Zigbee technology is based on the IEEE 802.15.4 standard [22] and guarantees (theoretically) a maximum transmission data rate of 250 kpbs over a wireless communication link. Three transmission bands are allowed by the Zigbee standard: (i) 2.4 GHz, (ii) 868 MHz, and (iii) 916 MHz. While the first transmission band is worldwide available, the second and third are available only in Europe and USA. In this paper, we focus only on the first two layers of the ISO/OSI stack (especially on the MAC layer): in this case, the Zigbee standard is equivalent to the IEEE 802.15.4 standard.

Since the communication between Zigbee nodes is on the same shared wireless medium, a MAC protocol is required to prevent collisions between data packets transmitted by different nodes. In particular, the IEEE 802.15.4 standard employs a non-persistent CSMA/CA MAC protocol. In addition, the IEEE 802.15.4 standard allows the use of an optional ACK message to confirm the correct delivery of a data packet. In a scenario with ACK messages, the access mechanism of the non-persistent CSMA/CA MAC protocol is slightly modified. While a generic data packet is sent according to the CSMA/CA protocol, an ACK message is sent back to the source immediately after the message is received by the destination node. If the source node does not receive the ACK message within a pre-fixed time interval, referred to as ACK window duration, the packet is declared lost and retransmitted. After three unsuccessful retransmission attempts, the packet is discarded and the node may start sending another data packet. As soon as the ACK message is received, the destination node (i.e., the node which has sent the data message and is waiting for the ACK message) waits for a period of time, referred to as long inter-frame spacing, which allows it to perform internal stack operations and process data (at the PHY layer). This interval is used also in the absence of ACK messages. In both cases, the receiving node, after sending the ACK message or receiving the data packet, waits for a shorter TAT, used to take into account radio frequency interface recalibration. During the TAT, the receiving node cannot accept new incoming packets.

We remark that the non-persistent CSMA/CA MAC protocol provides a medium access mechanism that tries to avoid packet collisions. Before transmitting a new packet, a node waits for an interval denoted as the backoff interval (*BI*). The backoff interval is randomly chosen within a range defined during the network start-up phase by the backoff exponent (*BE*) and expressed as a multiple of a reference time interval, which is referred to as the backoff unit and denoted as *T*_B_. In particular, the backoff interval is a random variable uniformly distributed in [0, (2*^BE^* − 1)*T*_B_]. For the first transmission attempt, the Zigbee standard defines *BE* = *BE*_min_ = 3. After the corresponding *BI* has elapsed, the node tries to send its packet again: if it detects a collision, it doubles the previously chosen maximum waiting interval (2*^BE^* − 1) and selects a new value for BI; if, instead, the channel is free, it transmits its packet. This procedure is repeated twice, after which, for the subsequent three unsuccessful transmission attempts, *BE* = *BE*_max_ = 5. After five unsuccessful retransmission attempts, the packet is dropped. This backoff algorithm makes it likely that a node will eventually manage to transmit its packet.

After the backoff period has expired, before effectively starting the packet transmission, a node needs to sense the channel in order to assess its status. The Zigbee standard provides a CCA technique which allows a node to sense the channel for a specific time interval, referred to as the CCA time. If at least another node transmits during this interval, the channel is declared busy and the node, which was sensing the channel, discards the packet and starts a retransmission.

### 3.2. Considered Opnet Model

**Figure 3.**
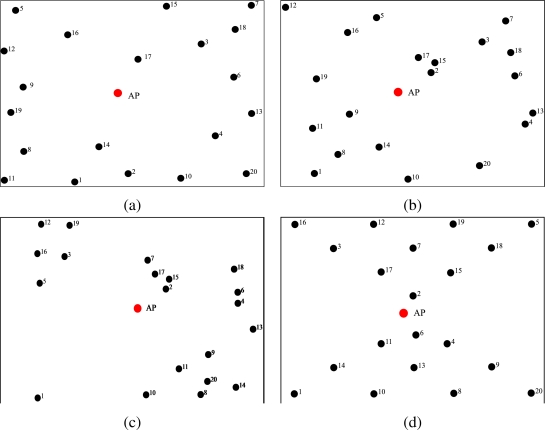
Considered network topologies with *N* = 20 nodes.

**Figure 4.**
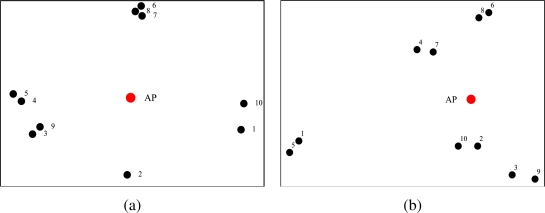
Considered network topologies with *N* = 10 nodes.

The simulations have been carried out with the Modeler package of the Opnet simulator [23] and a built-in Zigbee network model designed at the National Institute of Standards and Technologies (NIST) [24]. We have considered a scenario where *N* nodes transmit directly to the AP. In particular, the considered topologies for *N* = 20 are shown in Figure 3, whereas those for *N* = 10 are shown in Figure 4.

More precisely:

- in Figure 3a, *N* = 20 nodes are randomly deployed over a 100 m^2^ square area (the width of the side of the surface will become meaningful for the typical values of the transmit power considered in the following. Moreover, the maximum transmission range allowed by the Zigbee standard is 100 m) and are approximately concentrated towards the external perimeter of the surface (we point out that the considered surface for *N* = 10 sensors is smaller than that for *N* = 20 sensors);
- in Figure 3b, *N* = 20 nodes are deployed over the same surface as before, but present a few cluster and isolated nodes;
- in Figure 3c, *N* = 20 nodes are placed in order to form four small groups and only one node is isolated from the others;
- in Figure 3d, *N* = 20 nodes are placed over a regular grid and form two “triangular” grids which converge at the AP;
- in Figure 4a, *N* = 10 nodes are approximately at the same distance from the AP and form small groups isolated from each other;
- in Figure 4b, *N* = 10 nodes are clustered in groups of two. In particular, four pairs of nodes are placed near the AP, whereas the remaining pair is far from the AP.

We believe that the considered topologies are representative of a large set of possible WSN topologies. However, we remark that the proposed framework can be applied to a WSN with a generic topology.

**Figure 5.**
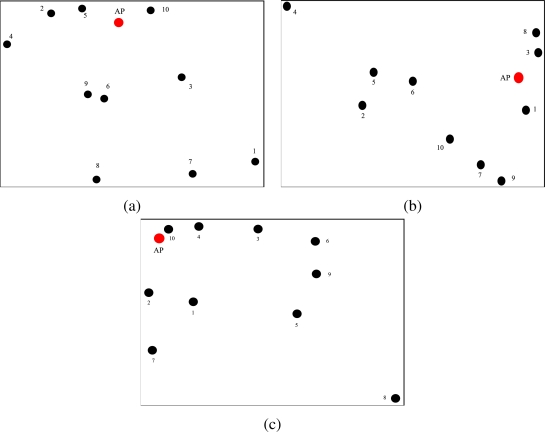
Network topologies with *N* = 10 nodes randomly deployed over a 10 m^2^ square surface, used for (a) PER comparison and (b) evaluation of the network lifetime. (c) Network topology with *N* = 10 nodes randomly deployed over a 50 m^2^ square surface, used for the evaluation of the network lifetime.

Since the proposed power allocation strategy aims at PER minimization, we have considered the network topology presented in Figure 5a to highlight the performance gain given by the proposed adjacency-based power allocation scheme. In order to highlight the impact of the proposed power allocation strategy on the network lifetime, we have considered two scenarios with *N* = 10 nodes randomly deployed over a 10 m^2^ square surface and over 50 m^2^ square surface. These topologies are shown in Figure 5b and Figure 5c, respectively.

Since the NIST Zigbee network Opnet model was developed to analyze the coexistence between IEEE 802.15.4 and IEEE 802.11 standards in small environments, it did not take into account signal attenuation [25]. In our simulations, instead, we have neglected the impact of co-existing IEEE 802.11 networks and we have introduced the channel attenuation according to the Friis propagation model. In particular, the Friis formula is given by Equation 1 and, in this paper, we assume *G*_r_ = *G*_t_ = 1 (omnidirectional antennas), *λ* = 0.125 m (*f*_c_ = 2.4 GHz), and *α* = 2.1. In all cases, *r* is shorter than 100 m, which is the maximum transmission range allowed by the Zigbee standard. If the received power is higher than a pre-defined threshold, fixed to −90 dBm, the nodes can exchange packets.

For each of the considered topologies, the distance between the nodes and consequently the power attenuation is computed offline on the basis of the coordinates of the nodes. These values are then used to fill the adjacency matrix. In particular, consider a pair of nodes (*s_i_, s_j_*) with *i* ≠ *j*: if *s_i_* is sufficiently close to transmit to *s_j_*, we insert a “1” in the corresponding entry of the adjacency matrix (i.e., the *i*th row and the *j*th column); otherwise, we mark the absence of communication with a “0”. We remark that the communication links may be asymmetric: even if *s_i_* can communicate with *s_j_*, the opposite may not hold. The distances between the nodes are also used to determine (i) the minimum (per-node) transmit power which allows each node to reach the AP and (ii) the maximum transmit power which guarantees that each node can reach any other node in the network.

The Zigbee standard provides indications about the values of the main network parameters introduced in Section 3.1. The values of the relevant parameters for our simulations are shown in Table 3. We remark that the Opnet simulator expresses all time-related parameters as multiples of the fundamental time unit, which corresponds to the inverse of the transmission data rate *R*. The simulations have been repeated several times with different seed initialization parameters in order to ensure that possible statistical fluctuations are avoided. The Opnet simulator also stores into log files the values of important metrics related to (i) packet transmission, such as the numbers of correctly received packets and noisy packets, and (ii) packet generation, such as the numbers of sent packets and dropped packets.

We remark that the simplified theoretical model presented in Section 2. is compliant with the simulation model just described.

## 4. Performance Analysis

In this section, we present the performance results in the presence of transmit power control. In particular, we focus on the following key performance indicators: the PER, the delay *D* (dimension: s), and the network transmission rate *S* (dimension: bit/s). The delay is defined as the average time interval between transmission and correct reception instants of a data packet. The network transmission rate is defined as the number of bits correctly received by the AP per unit of time. In addition, we present the performance results, in terms of residual energy, of the proposed power allocation strategy by comparing them with those obtained by the power allocation strategy proposed in [11].

The simulations have been carried out using different values of the overall network transmit power and, consequently, different values of the transmit powers allocated to the sensors. In particular, we have considered two possible transmit power allocation strategies: (i) each node has the same transmit power (uniform power allocation); (ii) the transmit power varies from node to node and is allocated using the strategy presented in Subsection 2.3.; (iii) the transmit powers are allocated according to the strategy proposed in [11]. In all cases, the obtained simulation results are directly compared with the results predicted by the theoretical model. In fact, referring to the scenarios shown in Figure 3 and Figure 4, we have first set the same transmission power at each node in order to allow (a) each couple of nodes to communicate with each other (the used transmit power is denoted as $P_{t}^{\text{max}}$) and (b) each node to reach at most the AP (the per-node transmit power is denoted as $P_{t}^{\text{min}}$). In the following, we will denote as {*P*_i_}, *i* = 1, . . ., *N* the transmit powers assigned to the nodes using the proposed power allocation strategies, and denote the overall available power as *P*_tot_. In particular, we will denote as $P_{\text{tot}}^{\text{max}}=P_{\text{tot}}^{\text{max}}/N$ the overall transmit power that guarantees that each node, using the same transmit power of $P_{t}^{\text{max}}=P_{\text{tot}}^{\text{min}}/N$, can transmit to any other node. Similarly, we will denote as $P_{\text{tot}}^{\text{min}}$ the overall power that guarantees that each node, using a transmit power of $P_{t}^{\text{min}}$, can reach at most the AP. We also remark that, in any case, the following condition will hold:

$$\sum_{i=1}^{N}P_{i}\leq P_{\text{tot}}$$

### 4.1. Validation of the Analytical Model

**Figure 6.**
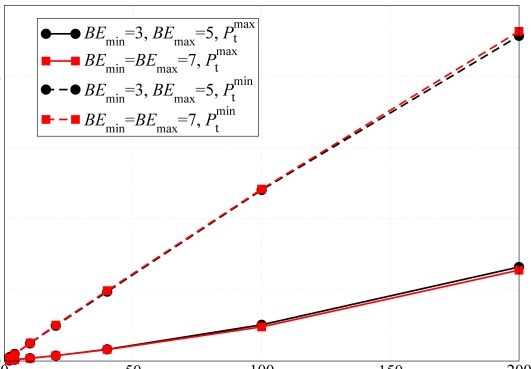
PER as a function of the total offered traffic load. The simulation results are obtained considering (i) default backoff exponent, i.e., *BE*_min_ = 3 and *BE*_max_ = 5, and (ii) modified backoff exponent, i.e., *BE*_min_ = *BE*_max_ = 7. Different values of per-node transmit powers are considered. The allocated transmit power is the same at each node.

Through simulations, we first validate the assumptions, behind the analytical model, of neglecting the impact of the backoff exponents on the network performance. We have compared the PER in a scenario with *N* = 20 nodes, different values of the backoff exponent and without the use of ACK messages. The results are shown in Figure 6. The solid lines refer to scenarios where each node can communicate with any other node in the network (the common per-node transmit power is $P_{t}^{\text{max}}$), whereas the dashed lines refer to scenarios where minimum common per-node transmit power (equal to $P_{t}^{\text{min}}$) is used. In both cases, the line with circles and the line with squares, which refer to scenarios with default and modified backoff exponents, basically overlap. This fact confirms the analytical assumption that the backoff exponent has a very limited impact on the network performance and it can be neglected, thus simplifying the theoretical model. A larger backoff exponent does not affect the performance in terms of PER, because the default value of the backoff exponent is large enough to decorrelate the backoff intervals of two nodes that could not transmit. In fact, according to the CSMA/CA MAC protocol described in Section 3.1., a node must double the range of the backoff interval and retry to transmit the packet after sensing the channel and finding it busy. If another node performs the same operations at the same time, some sort of correlation between the two transmitting nodes may emerge. However, the use of the random backoff interval guarantees that the two nodes will not collide at the subsequent transmission attempt.

**Figure 7.**
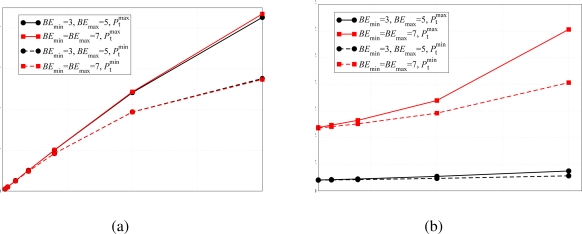
(a) Network transmission rate and (b) delay, as functions of the total offered traffic load (*Ng*), in the presence of (i) default backoff exponent, i.e., *BE*_min_ = 3 and *BE*_max_ = 5, and (ii) modified backoff exponent, i.e., *BE*_min_ = *BE*_max_ = 7. Different values of transmit powers are considered. The allocated transmit power is the same at each node.

The validity of the analytical model has also been verified in terms of delay and network transmission rate. The corresponding results are shown in Figure 7. As for the PER results in Figure 6, the network transmission rate is not affected by the use of different backoff exponent values. In fact, this performance indicator is strictly related to the PER, therefore, recalling the results shown in Figure 6, the network transmission rate remains basically the same for different values of *BE*_max_. Considering Figure 7a, the solid lines, which refer to the case with default backoff exponent, and the dashed lines, which refer to the case with modified backoff exponent, basically overlap. On the other hand, the use of different backoff exponents affects the delay performance. Since the backoff window is larger in the case with *BE*_min_ = *BE*_max_ = 7, a node may wait for a longer period before sending the packet on the channel. Considering Figure 7b, it can be observed that the delay is longer in the case with the modified backoff exponent. In fact, in this case a node has to wait, on average, for a longer period before transmitting a packet. In addition, using the transmit power $P_{t}^{\text{max}}$, the delay is higher because a node is more likely to sense other transmitting nodes during its CCA and, in this case, waits for a longer period before transmitting. On the other hand, if the common transmit power is set to $P_{t}^{\text{min}}$and no ACK mechanism is used, it is less likely that a transmitting node will sense another simultaneously transmitting (and thus colliding) node.

### 4.2. Impact of the Adjacency

**Figure 8.**
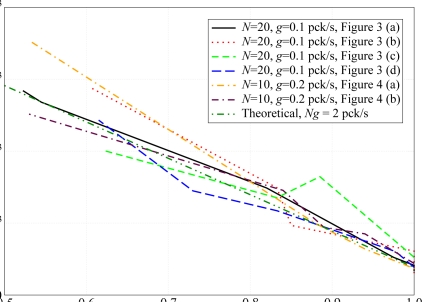
PER as a function of the sparsity index of the adjacency matrix. In the simulations, for scenarios with *N* = 20 nodes, the packet generation rate is set to *g* = 0.1 pck/s, whereas for scenarios with *N* = 10 nodes, the packet generation rate is set to *g* = 0.2 pck/s.

According to the analytical results in Section 2., the performance, in terms of PER, depends only on the adjacency, regardless of their specific positions. This emerges clearly from the results shown in Figure 8, where the PER is shown as a function of |*A*(*𝒮*)|*/N*^2^, i.e., the sparsity index of the adjacency matrix. In this figure, the performance with the network topologies in Figure 3 and Figure 4 is evaluated. For the simulation results, in the scenarios with *N* = 20 nodes, the packet generation rate is set to *g* = 0.1 pck/s, whereas in scenarios with *N* = 10 nodes, the packet generation rate is set to *g* = 0.2 pck/s, in order to keep the product *Ng* (i.e., the overall traffic load) constant and make the comparison between different topologies meaningful. In the same figure, the PER predicted by the analytical model, given by the expression in Equation 9, is also shown. As one can see, the simulation curves are very close to the corresponding analytical curves and this is more pronounced for values of |*A*(*𝒮*)|*/N*^2^ in the proximity of 1. In fact, when the value of |*A*(*𝒮*)|*/N*^2^ is close to 1, the network is strongly connected and a node can sense any other node. Observing the Opnet log files (not reported here for lack of space) stored by the nodes during the simulations, when |*A*(*𝒮*)|*/N*^2^ approaches 1 (i.e., the network is fully connected and during a CCA operation each sensor can detect the transmissions of all other sensors), the packets are dropped only during the TAT, when a node cannot sense other active nodes in the network during the transmission of its packets. On the other hand, when the value of |*A*(*𝒮*)|*/N*^2^ decreases, some nodes may become isolated from the other nodes (except for the AP) and may no longer be able to sense them, so that those packets may collide at the AP, leading to a PER increase.

Referring to Figure 8, the topology of the nodes in the the network has a very limited impact on the PER when |*Ā*(*𝒮*)|*/N*^2^ is close to 1 (the curves basically overlap). When |*Ā*(*𝒮*)|*/N*^2^ becomes lower, instead, the PER is higher in the scenarios relative to the topologies in Figure 3b and Figure 4a. For instance, considering node 12 in Figure 3b, when the transmit power is set to the minimum allowed value $P_{t}^{\text{min}}$ (equal at each node) to reach the AP, this node is isolated from most of the remaining nodes in the network. The packets transmitted by this node are likely to collide with those transmitted by nodes that are out of its transmission range, thus degrading the performance in terms of PER.

**Figure 9.**
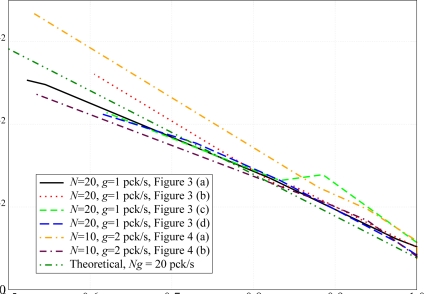
PER as a function of the sparsity index of the adjacency matrix. In the simulations, for scenarios with *N* = 20 nodes, the packet generation rate is set to *g* = 1 pck/s, whereas for scenarios with *N* = 10 nodes, the packet generation rate is set to *g* = 2 pck/s.

Similar considerations can be carried out for the scenarios where the aggregate traffic is set to *Ng* = 20 pck/s. The performance of these scenarios is shown in Figure 9. In this case, the statistical fluctuations are reduced since the number of packets transmitted by each node during the simulation is larger. However, the behavior in this case is also similar to that presented in Figure 8. There is a little dependence on the topology of the network, especially for values of |*Ā*(*𝒮*)|*/N*^2^ close to 1. When the number of ones in the adjacency matrix reduces, the PER increases and the impact of isolated nodes heavily affects the network performance.

### 4.3. Impact of Traffic

**Figure 10.**
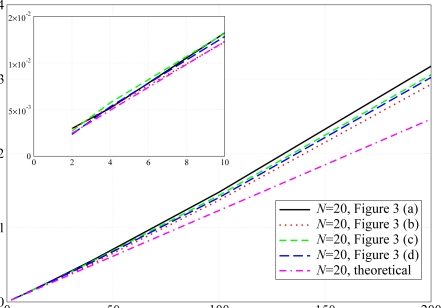
PER as a function of the aggregated offered traffic load *Ng*. Different topologies for scenarios with *N* = 20 nodes are considered. In all cases, the sparsity index is set to 0.87.

As shown in Section 2., the performance of a WSN, under the assumption of low traffic load, depends only on the number of ones in the adjacency matrix. In Figure 10, the sparsity index |*Ā*(*𝒮*)|*/N*^2^ is set to 0.87 and the PER is shown as a function of the aggregated offered traffic *Ng*. For all scenarios, we have considered at most *g* = 10 pck/s, since for larger values of *g* the assumption of low traffic load is no longer satisfied. The considered topologies are those in Figure 3. From our analysis it turns out that, for small values of *Ng*, all lines basically overlap, regardless of the network dimension and the number of nodes. In the inset of Figure 10, a closeup of the curves for low values of *Ng* is shown. The overlap of the curves is due to the proposed transmit power allocation strategy, which minimizes the number of collisions between the nodes. On the other hand, when *Ng* increases, the number of collisions increases as well, and the approximations behind the analytical model no longer hold. In this figure, the curve relative to the PER predicted by the analytical model and given by Equation 9 is shown. The PER predicted by the analytical model, for a given sparsity index, is lower than that obtained through simulations, especially for low offered traffic load. In our analytical model, in fact, we have assumed that under the assumption of low traffic load, the number of retransmission attempts due to the backoff algorithm is negligible. Through this transmit power allocation scheme it is then possible to set the transmit power at each node, in order to reach a desired sparsity index and consequently improve the performance.

**Figure 11.**
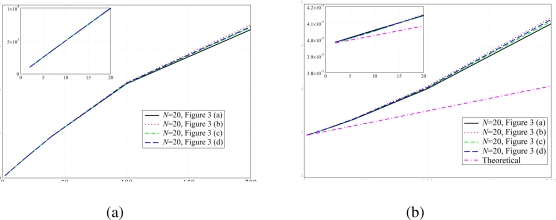
(a) Network transmission rate and (b) delay as functions of the aggregated offered traffic load *Ng*. Various topologies with *N* = 20 nodes are considered. The sparsity index is set at 0.87.

The same network configurations have been used in order to verify the impact of the sparsity index on the network transmission rate and delay. In Figure 11 a,b, these performance indicators are presented as functions of the overall traffic load. Considering Figure 11a, all presented curves basically overlap. This fact underlines once more that the number of ones in the adjacency matrix does not affect the performance. In particular, for small values of *Ng* the overlap is almost perfect and one can say that the performance depends only on the adjacency matrix and the related sparsity index. On the other hand, these results suggest that a given performance can be obtained with any network, provided that the transmit power is correctly allocated among the nodes.

Similar considerations can be carried out for the delay performance, analyzed in Figure 11b. In this case, there is also a good overlap between the simulation curves relative to different topologies and scenarios with different numbers of remote nodes. In particular, the number of ones in the adjacency matrix, i.e., the number of active connections, is the only characteristic that affects, for small values of the aggregated offered traffic load, the network performance. When the traffic load increases, however, the assumptions made in Section 2. do not hold anymore. In this figure, the analytical curve given by the expression in Equation 10 is also shown. In this case as well, the delay predicted by the analytical model is lower than that obtained with simulations. As in the previous case, the impact of the backoff procedure, due to the packet retransmission, has not been taken into account, thus the average delay predicted by our analytical model is lower.

### 4.4. Impact of the Power Allocation Strategy

In this subsection, we present the impact of the proposed transmit power allocation strategy on the performance of WSNs. In particular, as anticipated at the beginning of Section 4., we consider three possible transmit power allocation strategies.

1. In the former case, the transmit power is set in order to allow each sensor either to communicate with any other sensor in the network or to communicate at most with the AP.
2. In the proposed adjacency matrix-based power allocation strategy, the power is different at each sensor and is set according to optimization strategy presented in Section 2., where the total amount of available transmit power is assigned to each sensor in order to minimize the PER at the AP. This rule leads to allocate small transmit powers to nodes which are isolated and large transmit powers to nodes which can be connected with a large number of remote nodes. In this way, it is possible to minimize the collisions at the AP. Of course, there will still be nodes which cannot sense each other (leading to possible collisions), but this is due to the limited amount of overall network transmit power.
3. The power allocation strategy proposed in [11], which will be discussed in the following.

**Figure 12.**
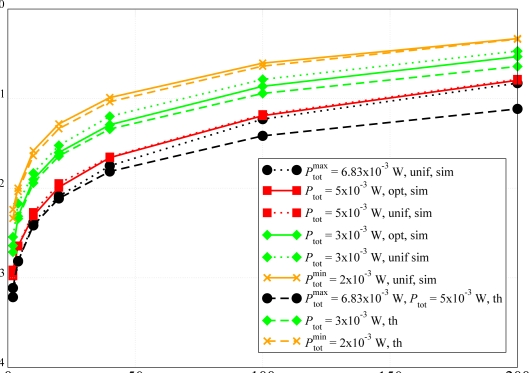
PER as a function of the aggregated offered traffic load *Ng*. Different transmit power allocation strategies are considered for the topology with *N* = 20 nodes presented in Figure 3b: (i) fixed per-node transmit power (unif) and (ii) optimized transmit power (opt). Both simulation (solid lines) and analytical (dashed lines) results are presented. The dotted lines refer to scenarios where no optimized power allocation strategy is used.

In Figure 12, the PER is shown as a function of the offered traffic load *Ng* in the network, for the topology presented in Figure 3b. Different values of overall network transmit power, with the corresponding sparsity indexes of the adjacency matrix shown in Table 4, are considered. Under the transmit power allocation strategy presented in Section 2., in Figure 12, a performance comparison between scenarios with and without the use of the proposed transmission power allocation strategy is presented. The overall transmission power available in the network is allocated either assigning a common transmit power to all nodes or using the proposed transmit power allocation strategy. Of course, in the latter case, the sparsity index of the adjacency matrix is maximized according to the available power and, referring to the results presented in Figure 8 and Figure 9, the higher is the sparsity index, the lower is the PER.

Fixing the sparsity index to 1, from the results in Figure 12 it can be observed that the performance is almost the same, regardless of the chosen transmit power allocation strategy. This confirms that the PER performance depends only on the number of ones in the adjacency matrix. In the other cases, when the number of connections between the nodes decreases, the probability of collisions at the AP increases, since it is likely that one transmitting node cannot sense another transmitting node out of its transmission range. However, the curves have the same trend for all values of offered traffic load. In particular, when *Ng* is low, it is likely that the number of collisions at the AP is low. Instead, when the traffic load is larger, the probability that two nodes transmit at the same time increases and the PER increases as well. In Figure 12, the analytical results are shown as dashed lines. These curves are close to those associated with simulation results, especially for scenarios in which the sparsity index is small. Once more, the good agreement between the analytical results and the simulation results is confirmed, thus further validating the analytical model. For the sake of comparison, in Figure 12 we also show the PER in scenarios where no transmit power allocation strategy is used (dotted lines). In these cases, the performance is worse than in the case with the optimized transmit power allocation strategy. In fact, given a value of overall network available power, the proposed power allocation strategy allows to maximize the sparsity index of the adjacency matrix and, therefore, reduce the PER.

**Figure 13.**
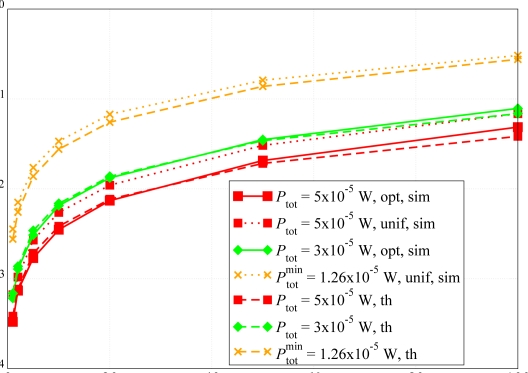
*P*_er_ as a function of the aggregated offered traffic load (*Ng*). Different power allocation configurations are considered for the topology with *N* = 10 nodes presented in Figure 4a. Both simulative (solid lines) and analytical (dashed lines) results are presented. The dotted lines refer to scenarios where no optimized power allocation strategy is used.

In Figure 13 the PER is shown as a function of the offered traffic load *Ng* for the scenario with *N* = 10 nodes presented in Figure 4a. The corresponding values of the sparsity indexes of the adjacency matrix are shown in Table 5. In this case, since the offered traffic load is lower, there is a better agreement between analytical and simulation models. In fact, the assumption of low traffic holds almost for all considered values of *Ng*. For small values of *Ng*, the curves are almost overlapped. Instead, when *Ng* increases, the backoff procedure leads to a gap between the simulation and the analytical curves. This gap, however, remains smaller than that in Figure 12. This confirms that the proposed analytical model can predict almost perfectly the performance of WSNs, especially for low values of *Ng*. Similarly to Figure 12, a comparison between the scenarios with (solid lines) and without (dotted lines) the use of the transmission power allocation strategy is also shown in Figure 13. In particular, given a pre-defined value of overall network available transmit power, the proposed approach maximizes the number of ones in the adjacency matrix and, consequently, improves the network performance.

**Figure 14.**
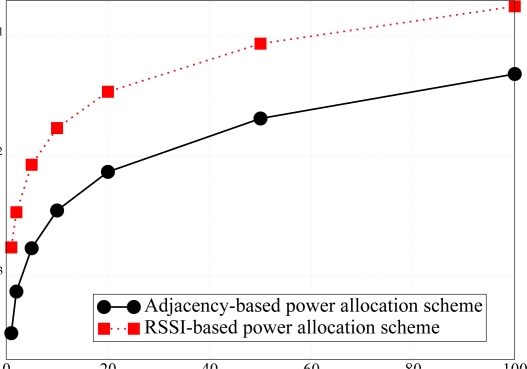
*P*_er_ comparison between the proposed power allocation scheme and the RSSI-based power allocation strategy presented in [11].

For the sake of completeness, a performance comparison between the PER guaranteed by the proposed power allocation scheme and that guaranteed by the power allocation scheme derived in [11] is considered in Figure 14. In [11], the authors aim at dynamically allocating the power at each node, in order to minimize the PER, under the assumption of additive white Gaussian noise (AWGN) channels and neglecting the impact of interference due to other transmitting nodes. In particular, they assume that the packet generation rate is low enough to prevent packet collisions. The power is allocated to each node according to the quality of the link that it experiences in order to reach the AP. The link quality is measured in terms of RSSI. In order to make the comparison fair, we have applied the power allocation strategy of [11] in a scenario without channel noise but in the presence of multiple access interference, which has been modeled as a Gaussian random variable [26]. As one can see from the results in Figure 14, our approach, based on the maximization of the sparsity of the adjacency matrix, tends to reduce as much as possible the number of collisions at the AP. Therefore, it is more efficient, especially for networks where the offered traffic load starts to become significant.

**Figure 15.**
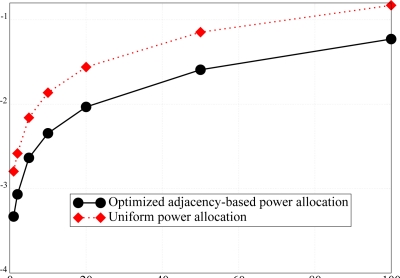
*P*_er_ comparison between the proposed power allocation scheme and the uniform transmit power allocation scheme.

In order to highlight the performance gain, in terms of PER, due to the proposed transmit power allocation scheme, with respect to a uniform power allocation strategy, the simulations have been carried out for a fixed overall network transmission power, considering the network topology presented in Figure 5a. The corresponding results, shown in Figure 15, underline that the proposed power allocation strategy effectively lowers the PER. The results in Figure 15 also suggest that, for a fixed target PER, the proposed power allocation scheme allows to support an almost double aggregated offered traffic load, with respect to that supported by a uniform power allocation strategy. In fact, through the proposed approach, a node, before transmitting, can sense a larger number of neighboring nodes (in a relatively dense partition of the network), and therefore prevent packet collisions.

### 4.5. Network Lifetime Performance

**Figure 16.**
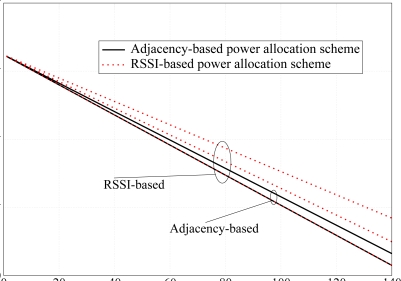
Residual energy per node. Both RSSI-based and adjacency-based transmit power allocation schemes are considered. The given PER is *P*_er_ = 5 · 10*^−^*^2^.

We now evaluate the network lifetime in the scenario with *N* = 10 nodes shown in Figure 5b, setting the average packet generation rate to *g* = 1 pck/s, and considering both the adjacency matrix-based power control approach proposed in this paper and the RSSI-based power control strategy presented in [11]. Since no battery models are provided in Opnet, the residual energy performance analysis has been carried out through Matlab. In particular, the battery depletion model refers to Equations 14–18. Each node is equipped with a 3 V battery with an initial energy of 32.4 kJ. In Figure 16, the residual energy per node is shown considering a target PER equal to *P*_er_ = 5 · 10*^−^*^2^. Since the distances between the nodes are small, the transmission powers of the nodes is low (between 0.374 *μ*W and 2.2 *μ*W). Since the current consumed during the transmission phase is *I*_tx_state_*i*_ = 7.886*P_i_* + 0.009711, the second term of the right-hand side of the current expression dominates, i.e., *I*_tx_state_*i*_ ≃ 0.009711. In other words, the effect of the transmit power on the energy consumed in the tx state is negligible. In fact, this is confirmed by the fact that the two families of curves, referring to adjacency-based (solid lines) and RSSI-based (dotted lines), are quite close. On the other hand, since the proposed approach aims at maximizing the connections between nodes, the power consumption is slightly higher in our approach than in [11].

**Figure 17.**
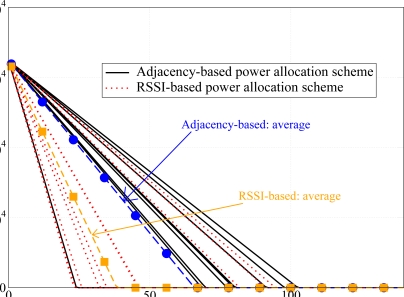
(i) Per-node (solid and dotted lines) and (ii) average network (dashed with symbols) residual energies as functions of time. Both RSSI-based (dotted lines and dashed with squares) and adjacency matrix-based (solid lines and dashed with circles) transmit power allocation schemes are considered. The target PER is *P*_er_ = 5 · 10*^−^*^2^.

In order to highlight the energy saving improvement introduced by our power allocation technique, we have considered the network, shown in Figure 5c, with *N* = 10 nodes randomly deployed over a 50 m^2^ square surface, where, unlike all previous scenarios, the path loss exponent *α* is 3 — recall that all previous results refer to scenarios with *α* = 2.1. In this case, the transmit power at each node is larger, so that the impact of the transmit power on the energy consumed in the tx state is more evident. In Figure 17, the residual energy in each node is shown, considering both the adjacency matrix-based transmit power allocation strategy proposed here and the RSSI-based power allocation scheme presented in [11]. Considering the proposed power allocation strategy (solid lines), the use of lower transmit powers allows to drastically reduce the nodes’ deaths. In particular, excluding the case of a specific node which is far from the AP and uses a high transmit power, the other nodes die later than in the case with RSSI-based power allocation scheme (dotted lines). In fact, according to the proposed model, a node delays its packet transmission if it senses that other neighboring nodes are transmitting. In this way, since a larger number of nodes can sense each other, the number of transmissions (successful or not) reduce and the nodes waste less power to process incoming packets. In the same figure, the average residual energy for both proposed power allocation schemes is also shown. These curves confirm that the adjacency-based power allocation scheme allows to extend the network lifetime because it increases the network residual energy.

**Figure 18.**
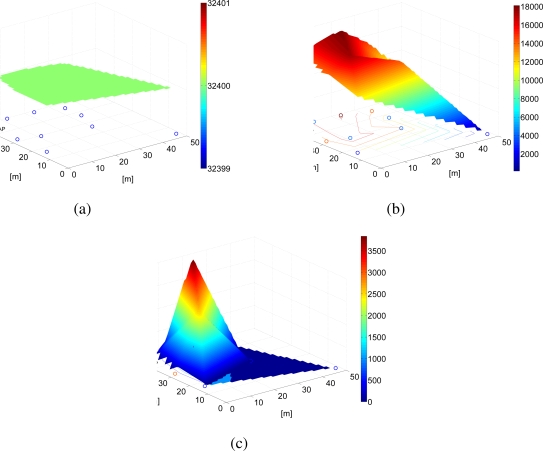
Residual energy with adjacency matrix-based transmission power allocation strategy proposed here, in a scenario with *N* = 10 nodes after (a) 0 days, (b) 45 days, and (c) 90 days.

Since each node transmits with a different power and receives a different number of packets from neighboring nodes, it will experience different power consumptions according to its spatial position and the number of surrounding nodes. In Figure 18, the energy distribution in the network considering the use of the proposed power allocation strategy is shown. The reference topology is the same as that considered in Figure 17 (i.e., the network topology of Figure 5c). In Figure 18a, the initial energy in the network, i.e., at all nodes, is shown. As one can see, since all nodes have the same battery energy (3.24 kJ), the initial “surface” lies on a plane. In Figure 18b, a snapshot of the residual energy in the network after 45 days is presented. The residual energy in the node far from the AP is lower than that in the other nodes, because this node transmits with much higher power. Finally, in Figure 18c, the residual energy in the network is presented after 100 days. The farthest node from the AP has run out of battery, but the other nodes are still able to communicate, since the use of low (on average) transmission powers prevents rapid battery depletion.

**Figure 19.**
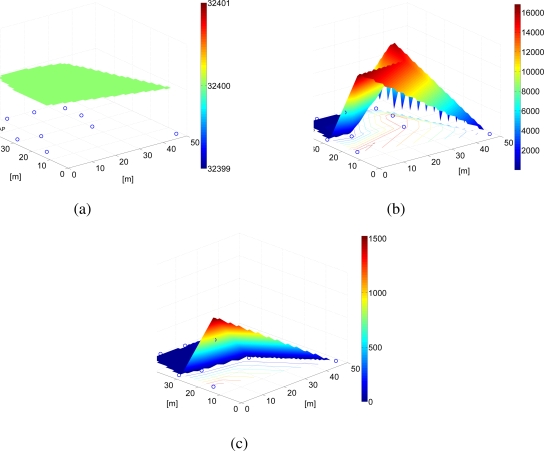
Residual energy with RSSI-based power allocation strategy of [11], in a scenario with *N* = 10 nodes after (a) 0 days, (b) 45 days, and (c) 90 days.

In Figure 19, the residual energy performance is shown considering the RSSI-based power allocation scheme presented in [11]. As in the previous case, in Figure 19a the initial energy in the network is shown (as for the previous figure, in this case as well all nodes have the same initial energy). In Figure 19b, the residual energy after 45 days is shown. As observed in Figure 18b, the farthest node from the AP has the lowest residual energy. However, in this case the nodes near the AP also have low residual energies. According to the RSSI-based power allocation scheme, in fact, these nodes experience low attenuation and are therefore assigned high values of transmit power. In this way, they consume a significant amount of energy to transmit a packet. Finally, in Figure 19c, the residual energy in the network after 100 days is shown. In this case, only one node is still alive, whereas the batteries of the remaining nodes have run out of energy.

## 5. Concluding Remarks

In this paper, we have presented an optimized transmit power allocation strategy which allows to minimize the PER at the AP of a WSN. First of all, we have derived a simplified analytical model which describes the performance of a Zigbee WSN, in terms of PER and delay, under the assumption of low offered traffic load. Then, we have presented the proposed transmit power control approach, developed under the assumption of finite overall network transmit power. In particular, we have shown that the performance basically depends on the number of ones in the adjacency matrix: this number represents the active connections between the nodes and is thus an index of the network connectedness. Our analytical model has been validated through the use of the Opnet simulator, underlying the impact, on relevant network performance indicators (PER, network transmission rate, delay, and network lifetime), of the sparsity index of the adjacency matrix, the offered traffic load, and the transmit power allocation strategy. In particular, we have verified that the proposed transmit power control approach, by maximizing the sparsity index of the adjacency matrix, allows to minimize the PER for a given total network transmit power, without reducing the lifetime of the network.

## Figures and Tables

**Table 1.** Current consumption in each state for a generic CC2420 radio module.

*I*_idle_state_	396 uA
*I*_rx_state_	19.6 mA
*I*_CCA_state_	19.6 mA
*I*_Boff_state_	396 uA
*I*_tx_state__*_i_*	7.886*P_i_* + 0.009711 mA

**Table 2.** Computation times for networks of different sizes. Results obtained with Mosek 5 (64-bit version) with a Core 2 Duo CPU at 3.16 GHz and with 4 GB RAM.

Number of sensors	Mean [s]	Std. Dev. [s]

10	0.040829	0.0064042
20	0.053216	0.00795104
50	0.17279	0.0730227
100	0.80042	0.336531
200	4.38015	1.46177

**Table 3.** Parameters of the Zigbee standard.

Fundamental time unit	4 *μ*s
LIFS	640 *μ*s
*T*_CCA_	128 *μ*s
ACK window duration	864 *μ*s
*T*_TAT_	192 *μ*s
*T*_B_	320 *μ*s
*L* (packet length)	512 (payload) + 120 (header) bits

**Table 4.** Sparsity indexes of the adjacency matrix for the scenario presented in Figure 3b. Different values of overall network transmit power are considered.

Available power (*P*_tot_)	Sparsity index
6:83 *·* 10^−3^ W $\left(P_{\text{tot}}^{\text{max}}\right)$	1
5 *·* 10^−3^ W	1
3 *·* 10^−3^ W	0.85
2:9 *·* 10^−3^ W	0.8325
2 *·* 10^−3^ W $\left(P_{\text{tot}}^{\text{min}}\right)$	0.605

**Table 5.** Sparsity indexes of the adjacency matrix for the scenario presented in Figure 4a. Different values of overall network transmit power are considered.

Available power (*P*_tot_)	Sparsity index
5 *·* 10^−5^ W	1
3 *·* 10^−5^ W	0.94
1:26 *·* 10^−5^ W $\left(P_{\text{tot}}^{\text{min}}\right)$	0.53
